# Clinical management of molecular alterations identified by high throughput sequencing in patients with advanced solid tumors in treatment failure: Real-world data from a French hospital

**DOI:** 10.3389/fonc.2023.1104659

**Published:** 2023-02-27

**Authors:** Sandra Pinet, Stéphanie Durand, Alexandre Perani, Léa Darnaud, Fifame Amadjikpe, Mathieu Yon, Tiffany Darbas, Alain Vergnenegre, Thomas Egenod, Yannick Simonneau, Valérie Le Brun-Ly, Julia Pestre, Laurence Venat, Frédéric Thuillier, Alain Chaunavel, Mathilde Duchesne, Véronique Fermeaux, Anne Guyot, Sylvain Lacorre, Barbara Bessette, Fabrice Lalloué, Karine Durand, Elise Deluche

**Affiliations:** ^1^ Medical Oncology Department, Dupuytren University Hospital, Limoges, France; ^2^ The National Institute for Health and Medical Research (INSERM) U1308 - CAPTuR “Control Of Cell Activation, Tumor Progression and Therapeutic Resistance”, Faculty of Medicine, University of Limoges, Limoges, France; ^3^ Cytogenetic, Medical Genetic and Reproductive Biology, Dupuytren University Hospital, Limoges, France; ^4^ Department of Pathology, Dupuytren University Hospital, Limoges, France; ^5^ Chest Department, Dupuytren University Hospital, Limoges, France; ^6^ Research Unit (UR) 20218 - NEURIT “Neuropathies et Innovations Thérapeutiques”, Faculty of Medicine, University of Limoges, Limoges, France

**Keywords:** cancer, FoundationOne CDx, next-generation sequencing, liquid biopsy, targeted therapy, precision oncology

## Abstract

**Background:**

In the context of personalized medicine, screening patients to identify targetable molecular alterations is essential for therapeutic decisions such as inclusion in clinical trials, early access to therapies, or compassionate treatment. The objective of this study was to determine the real-world impact of routine incorporation of FoundationOne analysis in cancers with a poor prognosis and limited treatment options, or in those progressing after at least one course of standard therapy.

**Methods:**

A FoundationOneCDx panel for solid tumor or liquid biopsy samples was offered to 204 eligible patients.

**Results:**

Samples from 150 patients were processed for genomic testing, with a data acquisition success rate of 93%. The analysis identified 2419 gene alterations, with a median of 11 alterations per tumor (range, 0–86). The most common or likely pathogenic variants were on *TP53*, *TERT*, *PI3KCA*, *CDKN2A/B*, *KRAS*, *CCDN1*, *FGF19*, *FGF3*, and *SMAD4*. The median tumor mutation burden was three mutations/Mb (range, 0–117) in 143 patients with available data. Of 150 patients with known or likely pathogenic actionable alterations, 13 (8.6%) received matched targeted therapy. Sixty-nine patients underwent Molecular Tumor Board, which resulted in recommendations in 60 cases. Treatment with genotype-directed therapy had no impact on overall survival (13 months *vs.* 14 months; p = 0.95; hazard ratio = 1.04 (95% confidence interval, 0.48–2.26)].

**Conclusions:**

This study highlights that an organized center with a Multidisciplinary Molecular Tumor Board and an NGS screening system can obtain satisfactory results comparable with those of large centers for including patients in clinical trials.

## 1 Introduction

Advances in molecular medicine have resulted in a shift in cancer treatment strategies from traditional therapies such as surgery, chemotherapy, and radiotherapy toward targeted therapy. A challenging issue in the field of cancer biology is to understand how cancers evolve, adapt, and resume growth after escaping specific treatments. Precision medicine, which has been proposed as the future of cancer treatment, is based on the understanding of genetic alterations as biomarkers of cancer (1). In the past 20 years, there has been a significant increase in European Medicines Agency (EMA)-approved molecular-targeted drugs for cancer treatment (2). For example, the identification of amplifications, translocations, and mutations in different cancer types, such as HER2 amplification in breast cancer, translocations in ALK- ROS1-or EGFR mutations in lung cancer, BRCA mutations in ovarian and breast cancer, and BRAF mutations in melanoma and lung carcinoma, have enabled the selection of specific treatments according to patient characteristics (3, 4). In the era of precision medicine in oncology, it is important to identify patients who are most likely to benefit from treatment, to optimize diagnostic testing and therapeutic follow-up, and to develop strategies to improve clinical efficacy. In this respect, diagnostic tests that comprehensively analyze genomic alterations in individual tumors are essential for successful delivery of personalized cancer therapy (5).

Recent advances in next-generation sequencing (NGS) have facilitated whole genome sequencing, whole exome sequencing, RNA sequencing, and development of large panels of target genes, as well as identification of a large number of genetic aberrations. NGS gene panels for cancer patients are used in daily clinical practice for the design and provision of personalized treatments by cancer centers worldwide. This is particularly important for cancer patients who have no approved treatment options. The European Society for Medical Oncology (ESMO) recently proposed different levels of recommendations for the use of NGS (5). Whole genome profiling can identify several oncological biomarkers to optimize patient treatment, especially in cases of relapse. If approved drugs targeting the genetic mutations detected are not available, patients can be included in clinical trials.

The FoundationOne^®^ CDx (F1CDx) test was approved by Food and Drug Administration (FDA) on November 30, 2017, under number P170019 (FDA), followed by the FoundationOne^®^ Liquid CDx (F1LCDx) based on circulating tumor DNA (ctDNA) analysis, which can be dedicated to patients with no available solid tissue corresponding to the disease stage. Next-generation *in vitro* (tissue-based) diagnostic tests, which rely on qualitative sequencing for cancer patients with advanced-stage solid tumors, are intended for prescription use only. These tests can analyze 324 genes and genomic signatures, as well as assess microsatellite instability (MSI) status and tumor mutation burden (TMB). Analytical and clinical validation for F1CDx/F1LCDx have been obtained to ensure the high sensitivity, specificity, and reliability of reported data (1, 6, 7).

Although NGS is increasingly used in routine management of targeted therapy options, few studies have assessed outcomes of patients following this test, and its feasibility in routine clinical practice. PROFILER-02, a multicenter randomized, prospective study, evaluated the proportion of metastatic cancer patients who received targeted agent recommendations based on two large NGS panels (8). According to the test used, the detection of abnormality genomic alterations varied between 5% and 31.9%, and only 15% of the patients initiated targeted treatment (8–10).

The department of this project, the Haute-Vienne, takes part in the Nouvelle-Aquitaine region with a low population density (67.5 inhabitants/km² compared to 121.6 inhabitants/km² at the national level (11). It has extensive rural areas, and the population is highly concentrated around our hospital (one in four inhabitants live in Limoges or its outskirts; source: Insee 2019). Patients are therefore confronted with two major problems: the difficulty of accessing care centres close to their homes for routine care and the difficulty of inclusion in clinical trials due to distance or individual wishes, which has been raised in the 2019 Cancer Plan: “Reduce inequalities and loss of opportunity”, in particular inequalities in health care due to the social and territorial situation (12).

The main objective was to investigate evaluate if an organized center with a multidisciplinary molecular tumor board and an organized NGS screening system can obtain satisfactory results comparable with those of large centers regarding inclusion of patients in clinical trials.

The secondary objectives were as follows: 1 to investigate the feasibility of clinical application of F1CDx and F1LCDx testing by evaluating the rate of failure of the test, 2, to evaluate the rate of detection of targetable or non-targetable abnormalities with the FMI test; 3, to evaluate the rate of targeted treatment based on molecular alterations identified by the FMI test; and 4, to evaluate the overall survival (OS) of patients who received targeted therapy according to the identified molecular alterations compared with that of the population that did not receive targeted therapy.

## 2 Materials and methods

### 2.1 Study and patient characteristics

On January 2018, the cancer center gained access to the NGS service platform, FMI, for analysis of solid tumor samples, and subsequently offered this service to patients with advanced disease. On January 2020 access to liquid biopsies was attained.

The inclusion criteria were as follows: Patients were considered candidates for FoundationOne CDx according to the oncologist’s decision and based on the clinical patient’s needs, i.e., cancer with a poor prognosis cancer and limited treatment options, or cancer in progression/recurrence after at least one course of standard therapy. There were no restrictions regarding tumor type or pathology. Patients should have18 years or older, with confirmed diagnosis of cancer, whether or not the cancers are stage IV, locally advanced cancer were accepted (i.e head and neck tumor, cervical cancer, vulvar cancer…) and available formalin-fixed paraffin-embedded (FFPE) tissue sample representative of the stage of the disease at the time of analysis/request for a FoundationOne^®^ CDx (F1CDx) test, or if there was no usable FFPE, the possibility to perform a liquid biopsy for FoundationOne^®^ Liquid CDx (F1LCDx). Exclusion criteria were: any medical or psychiatric condition which, in the Investigator’s opinion, would preclude the participant from giving consent to the test.

**Figure 1:**
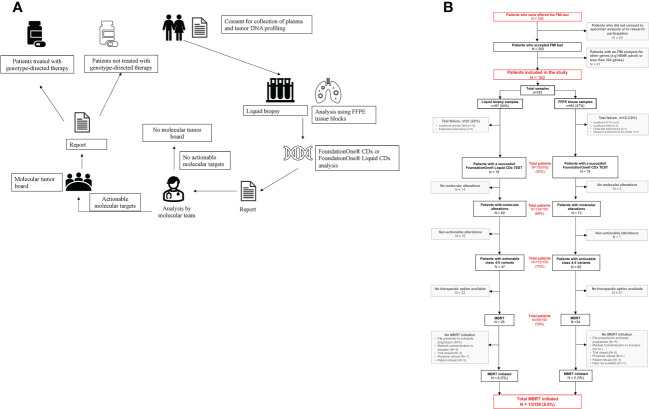
**(A)** Overview of our organization. **(B)** Study flow chart. F1LCDx, FoundationOne Liquid CDx; FCDX, FoundationOne CDx; FFPE, formalin-fixed paraffin embedded.

This prospective cohort study was conducted in a French hospital center to investigate the feasibility and utility of the clinical application of FMI testing. This project, named ClinMolCancer Study registration number: 87RI22_0043, include a total of 226 samples who were processed for genomic testing between 2018 and 2022. All patients included in the cohort complied with the regulations and provided signed consent for the molecular tests; the enrolled patients agreed to the use of care data for research purposes. An overview of the process is provided in **Figure 1A**.

### 2.2 Molecular analysis of solid tumors and liquid biopsies

After receiving the analysis request by the clinician and obtaining consent from the patient, the tumor library of the pathological anatomy department of Limoges University Hospital Center made the analysis fulfilled a test Requisition Form (TRF) on the FMI site. After receiving the analysis number (TRF number), the samples were collected as follows: FFPE samples containing representative tumor tissue were selected by a pathologist and sent directly to FMI. In case of uncontributive FFPE sample, liquid biopsies were collected on a Cell-Free DNA Collection Tube (Roche) by a nurse in the medical oncology department and transferred to the pathological anatomy department to be sent to FMI. FMI analyses consisted of genomic DNA (F1CDx) (7) and circulating cell-free DNA (cfDNA, F1LCDx) (6, 7) extraction, hybridization-based capture and sequencing of all coding exons of 309 genes, the intronic or non-coding regions of 21 of these genes, and selection of intronic or non-coding regions from an additional 15 genes, resulting in 324 total targeted genes. Additionally, MSI and TMB status, as well as the tumoral fraction for liquid biopsies, were reported. Sequence data were processed using a customized analysis pipeline designed to detect all classes of genomic alterations, including base substitutions, indels, copy number alterations (amplifications and homozygous gene deletions), and select genomic rearrangements (e.g., gene fusions). The data were received in the center by the biologists of the “Plateforme de Génétique Moléculaire des Cancers” (PGMC). The results were communicated in the form of a main report containing the known and likely pathogenic variants and suggestions for targeted therapeutics worldwide, and a supplementary data report containing all variants including Variants of Unknown Significance (VUS).

### 2.3 Use of molecular data in clinical practice: Multidisciplinary Molecular Tumor Board (MMTB)

The MMTB of the institution, which was established in 2019 (**Figure 1A**). At the MMTB meetings, which occur twice per month, molecular biologists, engineers of the PGMC, pathologists, and medical oncologists evaluate the therapeutic possibilities according to the highlighted molecular alterations and clinical patient status. Among MMTB, biologists and engineers of the PGMC classify the variants according to international variant classification databases (i.e., ClinVar, dbSNP, GnomAD, COSMIC, LOVD, Varsome, UMD, and OncoKB™) and the ESMO Scale for Clinical Actionability of Molecular Targets (ESCAT) recommendations (5, 13).

ESCAT classification or ESMO Scale of Clinical Actionability for provides a systematic framework to rank molecular targets based on clinical evidence of actionability (5, 13). It defines six levels of clinical evidence for molecular targets based on implications for patient management: targets implemented in routine clinical decisions (tier I); investigational targets that may define patients benefiting in a targeted drug but with the need for additional data (tier II); clinical benefit demonstrated in other tumour types or for similar molecular targets (tier III); preclinical evidence of actionability (tier IV); evidence supporting co-targeting approaches (tier V); and lack of evidence for actionability (tier X).

In addition, possible therapeutic approaches, including drugs with marketing authorization or those with early access in France, or those being tested by clinical trials in French centers, are selected. Cases showing therapeutically and clinically significant molecular alterations, including resistance mutations that may explain a poor response to previous therapeutic lines, are then reviewed by the MMTB, which recommends one of three possible molecular-based therapy options according to the patient’s medical file and the available clinical trial data: clinical trial, compassionate treatment, or conventional treatment.

### 2.4 Statistical analysis

This observational study was performed without determining sample size or power. Data were collected and analyzed using STATVIEW software version 5.0 (SAS Institute, Cary, NC, USA) and R software version 4.1.3 (R Foundation for Statistical Computing, Vienna, Austria). Quantitative results are expressed as the mean ± standard deviation or as the median (range), and qualitative results are expressed as numbers and percentages. Nominal variables were compared between groups using the Chi-square or Fisher’s exact tests, as appropriate. OS was defined as the time from the date of diagnosis of metastatic disease to the date of death (from any cause) or censored at the date of last contact. The Kaplan-Meier method was used to estimate OS, and log-rank tests were used to assess differences between patients treated with and without genotype-directed therapy. Survival differences between patients with low and high frequency of genetic alterations were also analyzed. The hazard ratio (HR) was estimated using a univariate Cox model and expressed with the 95% confidence interval (95% CI). Survival analyses were performed in R using the *survival* and *survminer* packages. Parameters with p value inferior to 0.2% in univariate Cox analysis were selected for multivariate Cox analysis. Number of individuals composing different group was indicated for each tested parameters.P-values < 0.05 were considered significant.

## 3 Results

### 3.1 Patient characteristics

The study flow chart is shown in **Figure 1B**. Of 226 patients with cytologically or histologically-confirmed advanced solid tumors who were offered the test, 203 agreed to the use of samples and associated data for research purposes.

Specimens from the 203 patients were considered adequate for genomic testing and were shipped to FMI. Of these patients, 41 were subsequently excluded from the study because the FMI test analyzed different genes (for example, the HEME panel) and/or less than 356 genes. The study cohort therefore included 162 patients; the characteristics of these patients (including the clinicopathological profiles) are summarized in **Table 1**. There were 81 women and 81 men, with a median age at diagnosis of 64 years (range, 19–84 years). Most patients (n = 142; 87.6%) had a good Eastern Cooperative Oncology Group performance status (0 or 1) at the time of testing. The most common tumor types were glioma (22%), lung cancer (18%), breast cancer (15%), and prostate and colorectal cancers (6%). The median delay between diagnosis and FMI test proposal was 2 years (range, 0–27 years).

**Table 1:** Patient characteristics.

Characteristic	Subset	No. of patients	%
**Total**		162	100
**Sex**	Female	81	50
	Male	81	50
**Median (range) age, years**		64 (19–84)	
**Type of cancer**	Glioma	36	22
	Lung cancer	29	18
	Breast cancer	25	15
	Colorectal cancer	10	6
	Prostate cancer	10	6
	Pancreatic cancer	9	6
	Ovarian cancer	7	4
	Head and neck cancer	6	4
	Kidney cancer	5	3
	Gastric cancer	5	3
	Biliary cancer	4	2
	Uterus cancer	4	2
	Cervical cancer	3	2
	Others brain tumor	2	1
	Esophageal cancer	2	1
	Small intestine cancer	1	1
	Skin cancer	1	1
	Sarcoma	2	1
	Anal cancer	1	1
**Stade**	Stade I-II-III	5	4
	Stade IV	119	96
**Grade**	II	4	11
	III	3	8
	IV	31	82
**ECOG PS**	0	45	28
	1	97	60
	2	13	8
	3	3	2
	Missing data	4	2
**Tissue source**	Biopsy	34	19
	Surgery	49	28
	Liquid biopsy	95	53
**Median (range) of sampling**		1 (1–3)	
**Delay between diagnosis and sampling (range), years**		2 (0–27)	
**Delay between sampling and results (range), days**		23 (6–142)	
**Number of previous lines of treatment**	0	9	6
	1	31	19
	2	46	28
	>2	73	45
	Missing data	3	2

### 3.2 Testing characteristics

The median delay between the date on which informed consent was obtained and the date of receipt of the NGS assay results by the treating physician was 23 days (range, 6–142 days).

The median number of tests per patient was 1 (range, 1–3); 17 patients had two or three samples because of the submission of non-contributory samples on the first test. In total, 181 samples were submitted to FMI, including 97 liquid biopsies (54%), and 84 FFPE tissues from 34 biopsies (19%) and 50 surgically resected tissues (28%) (**Figure 1B**).

Seventeen percent of the 181 samples (22% for the F1CDx test and 12% for the F1CDx test) were reported by FMI as failure or uncontributive tests: the F1LCDx test failed in 21 samples (22%) because the sample had insufficient cell-free DNA (n = 16), or the minimum performance was not achieved during the test (n = 5), whereas the F1CDx test failed in only 10 samples (12%) because percentage of tumor cells (n = 5) or DNA amount (n = 3) were insufficient, or because the sample did not achieve minimum performance during the test (n = 1), or it was not delivered to the center (n = 1) (**Figure 1B**).

Test failures affected most of the pathologies without distinction: among the most represented cancers, glioma testing failed in 22% (9/41) of cases, which were all liquid biopsy samples, and the reasons for failure were insufficient DNA quantity (n = 7) and to a lesser extent, performance during the test (n = 2); among lung cancer samples, testing failure occurred in 16% of cases (5/32), which were all liquid biopsy samples that failed because of insufficient DNA quantity (n = 3) or performance during the test (n = 2); in breast cancer, testing failure occurred in 14% (4/29) of cases, and the reasons for failure were insufficient DNA obtained from liquid biopsy (n = 1) or from surgically resected tissue (n = 2), or proportion of tumor cells under the limit of sensitivity for the test (n = 1). Testing failure was also observed in the following cancer samples: renal (n = 2), head and neck (n = 2), ovarian (n = 2), pancreatic (n = 1), biliary (n = 1), gastric (n = 1), esophageal (n = 1), colorectal r (n = 1), uterus (n = 1), and cervical (n = 1) cancers. A successful FMI test was achieved in 93% of the 162 patients.

The remaining 150 patients were analyzed for the presence or absence of molecular alterations to select appropriate therapies.

### 3.3 Prevalence of genetic variants and distribution of altered genes

When indicated by FMI reports, variants likely to originate from Clonal Hematopoiesis of Indeterminate Potential were excluded from the analysis.

**Figure 2:**
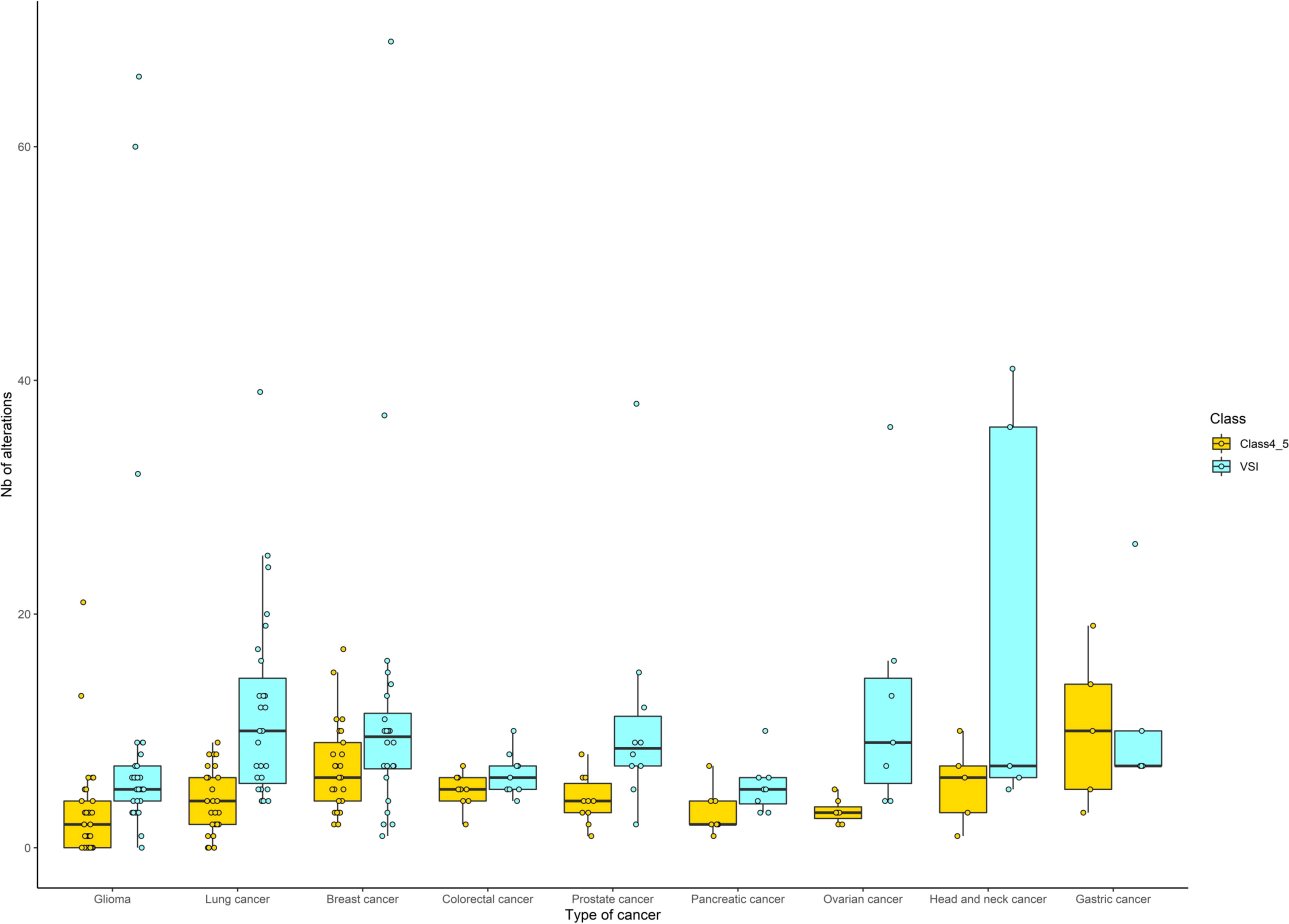
Distribution of the number of genetic alterations identified by sequencing (according to cancer type). For each tumor type, genetic alterations were classified into class 4/5 (corresponding to known and/or likely pathogenic) or VUS (corresponding to variant of unknown significance). Only tumor types with at least five cases were included and are presented according to decreasing number of patients: glioma (n=36), lung cancer (n=29), breast cancer (n=25), colorectal and prostate cancers (n=10), pancreatic cancer (n=9), ovarian cancer (n=7), head and neck cancer (n=6), and gastric cancer (n=5). Boxes indicate the median and quartile values, and points correspond to the number of alterations retrieved for each patient. Yellow or blue correspond to class 4/5 or VUS, respectively.

A total of 2419 alterations, with a median of 11 alterations per tumor (range, 0–86), were identified: 707 alterations were categorized as known/likely pathogenic variants (class 4-5) with a median of four alterations per tumor (range, 0–25), and 1712 alterations were VUS, with a median of seven alterations per tumor (range, 0–74). Globally, all tumor types had more VUS than class 4–5 variants. Glioma, pancreatic, and ovarian cancers had a lower proportion of class 4–5 variants and VUS, than lung and breast cancer. The proportion of VUS compared with class 4–5 variants is more important for glioma, prostate, ovarian, and head and neck cancers (**Figure 2**).

At least one variant was identified in 89% of all patients (n = 134/150), i.e., 82% (n = 62/76) and 97% (n = 72/74) of patients tested with F1LCDx and F1CDx, respectively (**Figure 1B**). Variant classification showed differences in variant prevalence according to the test used, with the F1CDx test identifying a greater number of variants: the F1LCDx test identified actionable class 4-5 variants in 62% (n = 47/76) of the patients, and non-actionable class 4–5 variants or VUS in 76% (n = 58/76 and n = 68/76) and 89% (n = 68/76) of the patients, respectively, whereas the F1CDx test identified actionable class 4–5 variants in 88% (n = 65/74) of the patients, and non-actionable class 4-5 variants or VUSs in 93% (n = 69/74) and 97% (n = 73/74) of the patients, respectively.

In addition, the number of actionable and non-actionable class 4-5 variants differed between the two tests according to pathology. Actionable class 4-5 variants were found in only 42% of gliomas compared with 66% of lung cancers, 90% of colorectal or prostatic cancers, and 96% of breast cancers. Non-actionable class 4–5 variants were identified in 61% of gliomas, 72% of lung cancers, 80% of prostatic cancers, and 100% of colorectal or breast cancers. VUS were found for all patients.

**Figure 3:**
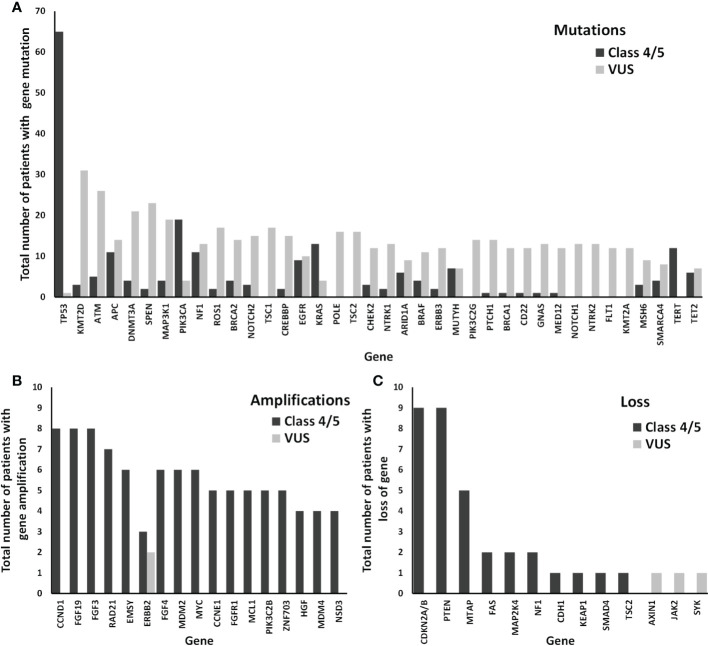
Distribution of the most frequently altered genes among our cohort of 162 patients. The numbers of patients with class 4/5 (known and/or likely pathogenic) and VUS (variant of unknown significance) genetic alterations are shown as black and grey bars, respectively: mutation **(A)**, amplification **(B)**, or loss **(C)**, of each gene.

Next, we analyzed the distribution of the most frequently altered genes in the cohort of 150 patients according to the type of alteration (mutation, amplification, or loss of copy number) (**Figure 3**). The median number of genes (with one or more variants) per patient was 3 (range, 0–19) for class 4–5 variants, and 7 (range, 0–61) for VUS. Among the genes altered by class 4–5 variants, TP53 was the altered most frequently, followed by PIK3CA, KRAS, PTEN, NF1, CDKN2A/B, TERT, and APC (**Figure 3**). Most altered genes with class 4–5 variants among representative cancer samples from the studied cohort are shown in **Table 2** and altered genes with all variants including class 4–5 and VUS are shown in **Supplementary Table 1**. The percentages of altered genes among representative cancers in our cohort are shown in **Table 3**. TP53 was the only gene that was altered in all representative cancer subtypes in our cohort. Regarding VUS, the most frequently altered genes were ATM, KTMT2D, SPEN, NOTCH2, and DNMT3A (**Supplementary Table 2**). Only genes with class 4–5 variants were considered for subsequent analyses.

**Table 2:** Most altered genes among representative cancers in our cohort (number of patients, along with genetic alterations and frequency).

Cancer type	Genes	Nb	Freq. (%)	Cancer type	Genes	Nb	Freq. (%)
**Glioma (n=33)**	**TP53**	8	24	**Prostate (n=10)**	**TP53**	6	60
	**TERT**	8	24		**AR**	4	40
	**PTEN**	7	21	**Pancreatic (n=8)**	**CTNNB1**	2	25
	**NF1**	6	18		**KRAS**	2	25
	**EGFR**	5	15		**SMAD4**	2	25
**Lung (n=27)**	**TP53**	14	52		**TP53**	2	25
	**KRAS**	4	15	**Ovarian (n=7)**	**TP53**	6	86
**Breast (n=25)**	**PIK3CA**	9	36		**PIK3R1**	2	29
	**ESR1**	7	28	**Head & Neck (n=6)**	**TP53**	3	50
	**C11orf30 (EMSY)**	6	24		**CCND1**	2	33
	**TP53**	6	24		**CDKN2A/B**	2	33
	**CCND1**	5	20		**DNMT3A**	2	33
	**FGF19**	5	20		**FGF19**	2	33
	**FGF3**	5	20		**FGF3**	2	33
	**FGF4**	5	20		**TERT**	2	33
	**FGFR1**	5	20	**Gastric (n=5)**	**TP53**	3	60
	**ZNF703**	5	20		**ARID1A**	2	40
	**CDH1**	4	16		**ATM**	2	40
	**NSD3 (WHSC1L1)**	4	16		**CDKN2A/B**	2	40
**Colorectal (n=10)**	**TP53**	8	80		**PIK3CA**	2	40
	**APC**	6	60				
	**KRAS**	3	30				
	**SMAD4**	3	30				

Cancers for which more than five patients had available sequencing results were analyzed. Genes with class 4-5 variants in at least four patients with the most represented cancers (glioma, lung, and breast cancers), and those with class 4-5 variants in at least two patients with the least frequent cancers (colorectal, prostate, pancreatic, ovarian, head and neck, and gastric cancers), were selected for the global cohort. Abbreviations: Nb., number of patients with genetic alterations; Freq., frequency (in %).

**Table 3:** Percentage of altered genes among representative cancers in our cohort.

Type of cancer	Glioma (n=33)	Lung (n=29)	Breast (n=25)	Colorectal (n=10)	Prostate (n=10)	Pancreatic (n=8)	Ovarian (n=7)	Head & Neck (n=6)	Gastric (n=5)
**TP53**	24	52	24	80	60	25	86	50	60
**KRAS**		15		30		25			
**TERT**	24							33	
**CCND1**			20					33	
**FGF19**			20					33	
**FGF3**			20					33	
**PIK3CA**			36						40
**SMAD4**				30		25			
**CDKN2A/B**								33	40
**PTEN**	21								
**NF1**	18								
**EGFR**	15								
**ESR1**			28						
**C11orf30 (EMSY)**			24						
**FGF4**			20						
**FGFR1**			20						
**ZNF703**			20						
**CDH1**			16						
**NSD3 (WHSC1L1)**			16						
**APC**				60					
**AR**					40				
**CTNNB1**						25			
**PIK3R1**							29		
**DNMT3A**								33	
**ARID1A**									40
**ATM**									40

Cancers for which more than five patients had available sequencing results were analyzed. The numbers indicate the frequency of class 4-5 variants among cases of cancer; Yellow = <30%; orange = ≥30% and <50%; and red = ≥50%.

### 3.4 Biological pathways

A listing of 324 genes included in the F1CDx and F1LCDx panels was submitted to DAVID (Database for Annotation, Visualization and Integrated Discovery), and the enrichment tool was used for retrieving KEGG pathways for each gene. We identified 25 pathways associated with common signaling pathways that are dysregulated in cancer (**Supplementary Table 3**). Fewer pathways were associated with gliomas (and to a lesser extent prostate and head and neck cancer) than with other cancers such as breast, colorectal, ovarian, and gastric cancers (**Table 4**).

**Table 4:** Altered pathways (KEGG) in different types of cancer.

			% samples per pathway alteration								
KEGG ID	KEGG pathway	Nb of genes	Glioma (n=33)	Lung (n=27)	Breast (n=25)	Colorectal (n=10)	Prostate (n=10)	Pancreatic (n=8)	Ovarian (n=7)	Head & Neck (n=6)	Gastric (n=5)
**hsa05200**	**Pathways in cancer**	112	54,5	74,1	96,0	100,0	100,0	62,5	100,0	66,7	100,0
**hsa04151**	**PI3K-Akt signaling pathway**	73	51,5	66,7	88,0	100,0	60,0	62,5	100,0	66,7	100,0
**hsa01521**	**EGFR tyrosine kinase inhibitor resistance**	38	42,4	37,0	80,0	60,0	30,0	37,5	57,1	16,7	80,0
**hsa04010**	**MAPK signaling pathway**	54	48,5	66,7	92,0	100,0	70,0	37,5	100,0	66,7	100,0
**hsa04012**	**ErbB signaling pathway**	30	27,3	40,7	72,0	60,0	30,0	25,0	42,9	33,3	80,0
**hsa04014**	**Ras signaling pathway**	44	39,4	33,3	88,0	70,0	20,0	25,0	57,1	33,3	80,0
**hsa05235**	**PD-L1 expression and PD-1 checkpoint pathway in cancer**	26	36,4	37,0	60,0	60,0	20,0	37,5	42,9	33,3	60,0
**hsa04630**	**JAK-STAT signaling pathway**	31	24,2	22,2	68,0	30,0	20,0	25,0	42,9	33,3	80,0
**hsa04150**	**mTOR signaling pathway**	25	30,3	37,0	64,0	70,0	20,0	37,5	42,9	16,7	80,0
**hsa04310**	**Wnt signaling pathway**	16	27,3	59,3	48,0	100,0	70,0	62,5	85,7	50,0	60,0
**hsa04340**	**Hedgehog signaling pathway**	9	0,0	0,0	24,0	0,0	0,0	25,0	0,0	33,3	0,0
**hsa04330**	**Notch signaling pathway**	6	3,0	0,0	4,0	0,0	0,0	0,0	0,0	0,0	40,0
**hsa04064**	**NF-kappa B signaling pathway**	11	3,0	7,4	8,0	0,0	0,0	12,5	0,0	16,7	40,0
**hsa04722**	**Neurotrophin signaling pathway**	27	36,4	59,3	72,0	90,0	70,0	37,5	100,0	50,0	80,0
**hsa04390**	**Hippo signaling pathway**	18	3,0	11,1	44,0	80,0	10,0	50,0	0,0	33,3	80,0
**hsa04115**	**p53 signaling pathway**	21	42,4	51,9	60,0	80,0	70,0	62,5	85,7	66,7	80,0
**hsa04110**	**Cell cycle**	27	36,4	51,9	60,0	90,0	60,0	62,5	85,7	66,7	80,0
**hsa04210**	**Apoptosis**	27	33,3	55,6	64,0	90,0	60,0	50,0	100,0	66,7	80,0
**hsa05202**	**Transcriptional misregulation in cancer**	39	30,3	59,3	36,0	100,0	70,0	37,5	100,0	66,7	80,0
**hsa03440**	**Homologous recombination/alterations in the DNA repair pathway**	16	6,1	0,0	16,0	0,0	0,0	25,0	14,3	16,7	40,0
**hsa03430**	**Mismatch repair**	6	6,1	3,7	4,0	0,0	0,0	0,0	0,0	16,7	0,0
**hsa03410**	**Base excision repair**	6	6,1	3,7	12,0	0,0	0,0	0,0	14,3	0,0	0,0
**hsa01524**	**Platinum drug resistance**	22	36,4	51,9	60,0	80,0	60,0	37,5	100,0	66,7	80,0
**hsa04120**	**Ubiquitin mediated proteolysis**	12	12,1	18,5	16,0	10,0	0,0	0,0	0,0	0,0	40,0
**hsa04140**	**Autophagy - animal**	23	30,3	33,3	64,0	60,0	10,0	37,5	42,9	16,7	80,0

Cancers for which more than five patients had available sequencing results were analyzed. Pathways associated with 324 genes from the FoundationOne CDx panel were retrieved from the KEGG database using the DAVID (Database for Annotation, Visualization and Integrated Discovery) enrichment tool. The percentage of patients with 4-5 alterations in at least one gene belonging to a KEGG pathway are indicated in a yellow-to-red gradient (yellow = <30%; orange = ≥30% and <50%; and red = ≥ 50%).

### 3.5 ESCAT and prognostic values

**Table 5** provides a list of actionable and biologically relevant alterations detected in our cohort based on ESCAT (5). Among the cohort patients, 14% harbored at least one alteration belonging to tier I (n= 22), 8.9% had alterations belonging to tier II (n = 14), and 25% had alterations belonging to tier III (n = 39). The most frequently found ESCAT I alteration was a PI3CKA mutation in breast cancer, followed by RET fusion alterations in lung cancer and MSI high for all cancers. The most frequent ESCAT II alteration was the *ESR1* mutation in breast cancer.

**Table 5:** Classification based on ESCAT I, II, and III of class 4-5 variants identified by sequencing in our cohort.

ESCAT	Alterations	Type of tumor	Total no.	Alteration prevalence (%)
**Tier I**	**Target suitable for routine use with a recommended drug when a specific molecular alteration is detected**			
	ERBB2 amplification	breast cancer, gastric cancer	2	6.7
	PIK3CA mutation	breast cancer	10	40
	BRCA 2 somatic mutation	ovarian cancer, prostate cancer	1	5.9
	EGFR common mutation	lung cancer	0	0
	ALK fusions	lung cancer	1	3.7
	ROS1-rearranged	lung cancer	1	3.7
	NTRK gene fusions	all cancer	0	0
	MET mutation	lung cancer	0	0
	RET fusion	lung cancer	3	11
	MSI-H	all cancer	3	2.2
	FGFR2 fusion	cholangiocarcinoma	0	0
	IDH mutation	cholangiocarcinoma	0	0
	BRAF V600E mutation	melanoma, colorectal cancer, lung cancer	1	2.6
**Tier II**	**Investigational targets likely to define patients who would benefit from a targeted drug, but additional data is needed**			
	PTEN mutations	breast cancer, prostate cancer	1	2.9
	AKT mutations	breast cancer	2	8
	ERBB2 mutations	breast cancer. Lung cancer; gastric cancer	0	0
	ERBB2 amplification	colorectal cancer	0	0
	ESR1 mutations	breast cancer	7	28
	KRAS mutations	lung cancer	4	14.8
	ATM mutations/deletions	prostate cancer	0	0
	PALB2 mutations	prostate cancer	0	0
	MET amplification	gastric cancer	0	0
	BRAF V600E mutation	cholangiocarcinoma	0	0
	EGFR amplification	gastric cancer	0	0
**Tier III**	**Clinical benefit previously demonstrated in other tumor types or for similar molecular targets**			
	*ERBB3 mutations*	*breast cancer, gastric cancer*	1	3.3
	ATM mutations	colorectal cancer, gastric cancer	4	26.7
	MET amplification	colorectal cancer, cholangiocarcinoma	0	0
	MET mutation	gastric cancer	0	0
	AKT mutations	colorectal cancer, prostate cancer	0	0
	PIK3CA, PIK3CB, PIK3CD	all cancer except breast cancer	13	10.2
	BRAF V600E mutation	all cancer except melanoma, colorectal cancer, lung cancer, cholangiocarcinoma	0	0
	FGFR amplification	all cancer	5	3.3
	MDM2 amplification	breast cancer, pancreatic cancer	1	3
	PIK3CA mutation	all cancer except breast cancer	12	9.4
	NRG1 fusion	lung cancer, pancreatic cancer	NA	NA
	RET fusion	colorectal cancer	0	0
	BRCA 2 somatic mutation	lung cancer, gastric cancer, pancreatic cancer, breast cancer	3	4.6
**Not applicable because of resistance biomarker**				
	NF1	breast cancer	3	12
	STK11	lung cancer	3	11
	KRAS and NRAS mutation	colorectal cancer	4	40

NB: PIK3CD and NRG1 were not available in the set of sequencing genes. NA, Not applicable.

**Figure 4:**
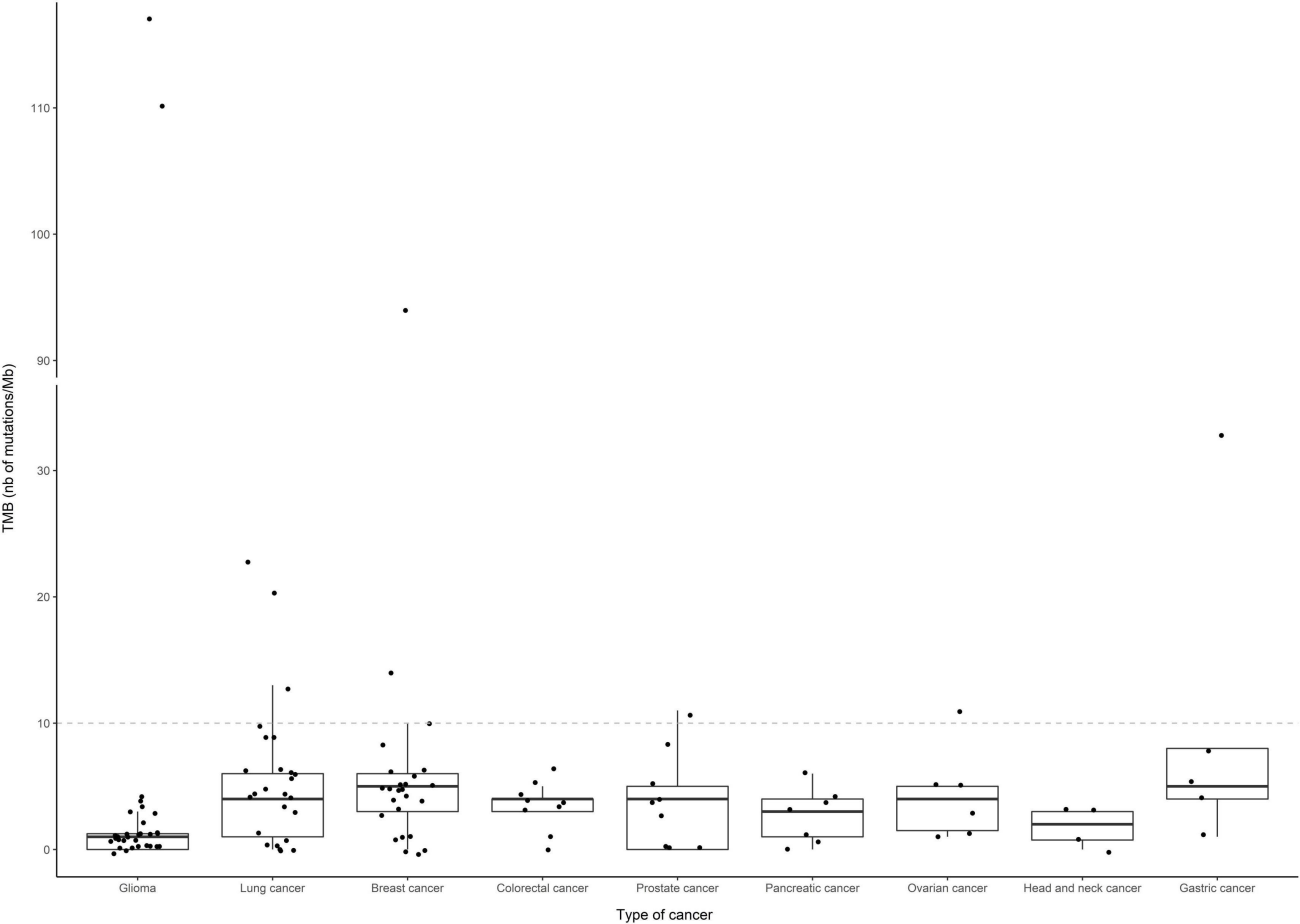
Distribution of TMB (number of mutations per megabase) according to the type of cancer. Only tumor types with at least five cases with sequencing results were included in the representation, and are listed by decreasing number of patients: glioma (n=36), lung cancer (n=29), breast cancer (n=25), colorectal and prostate cancers (n=10), pancreatic cancer (n=9), ovarian cancer (n=7), head and neck cancer (n=6) and gastric cancer (n=5). Boxes indicate the median and quartile values, and points correspond to individual TMB values. A dashed grey line indicates the cutoff (10) for high and low TMB values.

Significant differences in OS were observed after classifying patients according to the number of class 4–5 variants, regardless of cancer type. Patients with none, one, or two class 4–5 variants (n = 64) had a median survival of 16 months (95% CI, 13.0–NA), whereas patients with three or more class 4–5 variants (n = 88) had a median survival of 10 months (95% CI, 7.0–18.0) (p = 0.024) (**Figure 4**). A high frequency of class 4–5 variants (≥3) had an HR of 1.8 (95% CI, 1.07–3.02, p = 0.026) in the univariate Cox regression model.

### 3.6 Prevalence of MSI and TMB

Among the 150 patients assessed for MSI, 3 (2.2%) were categorized as MSI-high, 70 (47%) as MSI stable, 65 (43%) as MSI-high not detected and the remaining 12 (8%) as “cannot be determined”. No difference in OS was observed between patients stratified by MSI or MSS status (p = 0.81; data not shown).

In 143 patients with available TMB results, the median TMB was 3 mutations/Mb (range, 0–117 mutations/Mb. Although there were two patients with excessively high TMB among the gliomas, this subtype of cancer had the lowest median TMB (one mutation/Mb) among the most represented cancers in our cohort (**Figure 4**). After setting the TMB threshold to 10, patients were classified into negative, low, and high TMB groups comprising 27, 98, and 17 patients, respectively. There were no differences in OS between patients stratified by TMB status (p = 0.36; data not shown).

### 3.7 MMTB organization and proportion of patients treated with genotype-directed therapy

The test was ordered following an organ-dedicated multidisciplinary tumor board (n = 33, 20.4%) or following a medical decision alone (n = 124, 76.54%). The median deadline for performing the MMTB test after the date in which the results of F1CDx and F1LCDx panels were obtained was <1 month (range, 0–6).

The proportion of patients evaluated by the MMTB to assess the clinical utility of the FMI tests was determined. Among the 162 tested patients and the 150 with a contributive FMI test, 112 had at least one actionable known (class 5) or likely pathogenic (class 4) alteration. MMTB assessments were performed in 69 patients and resulted in therapy recommendations: compassionate treatment in 11 patients and a clinical trial in 50 patients. Finally, 13 of these patients (8.6%) received the corresponding targeted therapy (**Figure 1B**), of whom 5% were tested by liquid biopsy and 3% by FFPE tissue analysis.

Reasons for non-compliance with the MMTB were as follows: patient decision (n = 8, 13%), physician decision (n = 1, 1.6%), trial not accessible in the clinical trial center (n = 16, 26.2%), not applicable because the result was requested in anticipation of recurrence (n = 12, 19.0%), and clinical contraindication to inclusion in a clinical trial or palliative care (n = 19, 31.1%). Seven patients died before the results were obtained.

The median follow-up period after testing for the whole cohort was 7 months (range, 0–43 months). The median OS was 14 months (95% CI, 11–19).

**Figure 5:**
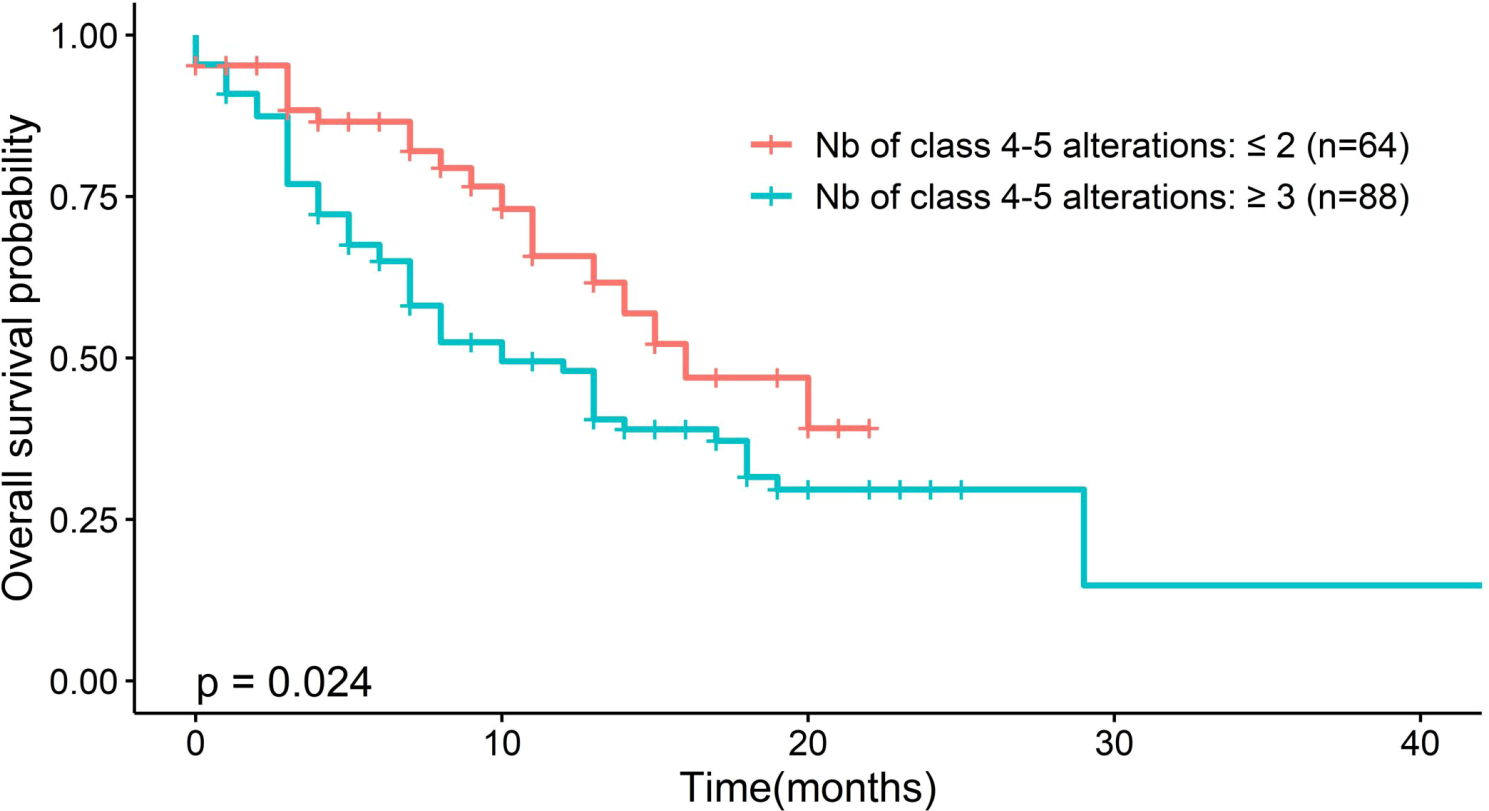
Association between the frequency of class 4-5 gene alterations and survival rates. Kaplan-Meier curves are depicted according to low frequency of genetic alterations (none, one, or two altered genes per patient) versus high frequency (more than three altered genes per patient). P-values were determined by the log-rank test and used to compare two survival curves.

**Figure 6:**
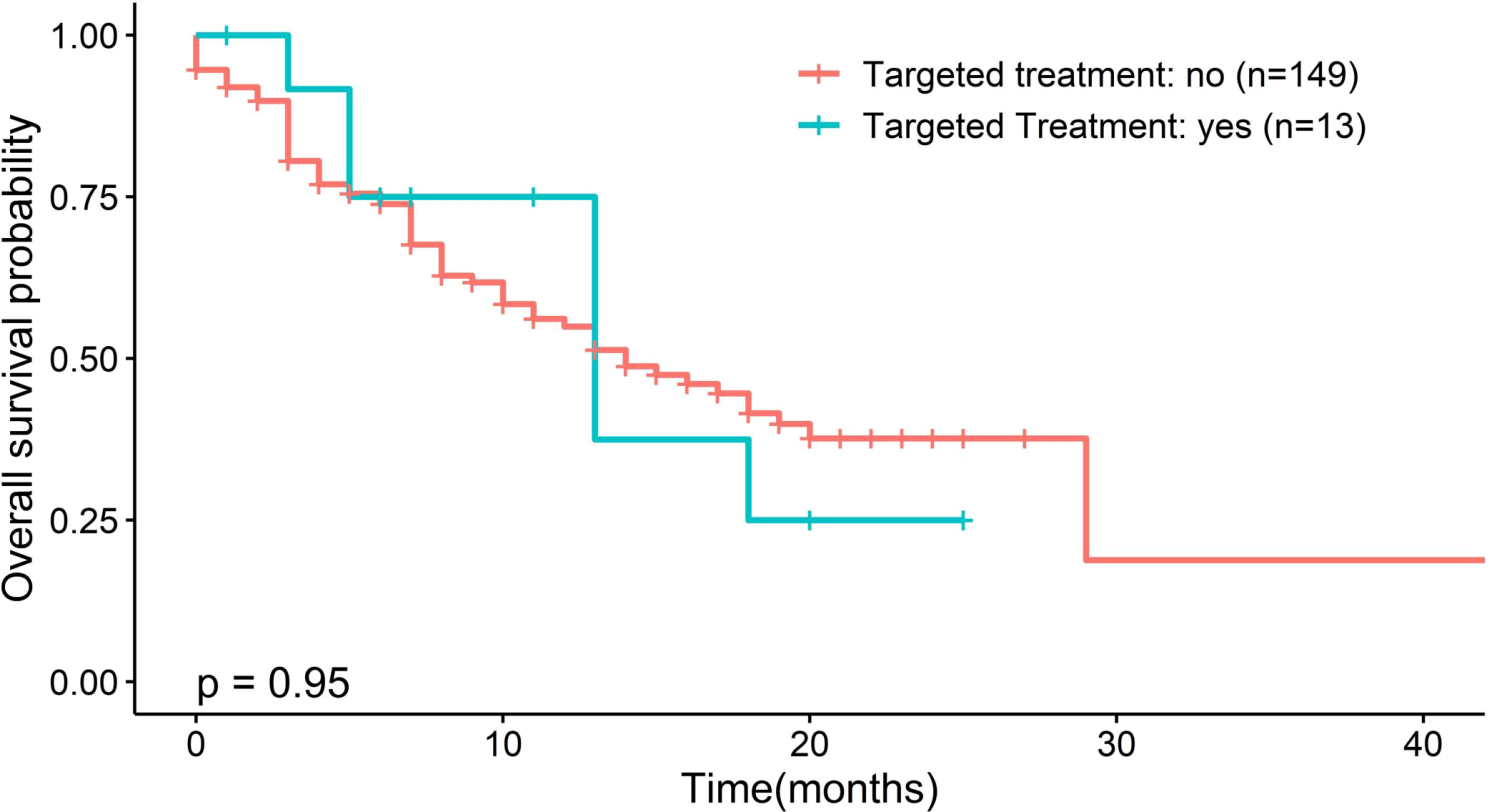
Kaplan-Meier plots of overall survival comparing patients treated with and without genotype-directed therapy.

In univariate and multivariate analyses, chemotherapy, performans status and Number of class 4-5 variants were significantly associated with OS (P < 0.05) (**Figure 5** and **Supplementary Figure 1**). The median OS did not differ significantly between patients treated with genotype-directed therapy (13 months, 95% CI, 13.0–NA) and patients who were not treated with genotype-directed therapy (14 months, 95% CI 11.0–20.0, p = 0.95 HR = 1.04 (95% CI, 0.48–2.26) (**Figure 6** and **Supplementary Figure 1**).

## 4 Discussion

Foundation One CDx assays can identify a large number of mutations, including actionable mutations that can be targeted by new therapies, as well as measure genomic signatures and clinical biomarkers, such as TMB and MSI which are potential predictive biomarkers for immunotherapy.

Here, we evaluated, the value and therapeutic impact of these tests in a large number of cancer samples in real life, and independent from inclusion in clinical trials. To our knowledge, this is the first real study conducted in a university centre without early phase trials (compared publications from comprehensive cancer centre). This highlights that other types of centres may also have a place in the implementation strategy of molecular screening. This is particularly important in regions that are under-resourced in terms of medical resources, where the population is far from health centres. The results of the study indicated that an extended molecular screening of samples obtained from tumor biopsies and/or liquid biopsies was feasible in almost all of the patients included (93%). Genomic alterations were actionable in 75% of patients with advanced, heavily pretreated malignant diseases. This high success rate suggests that such sequencing is relevant in clinical practice. The success rate was 78% for liquid biopsy samples and 88% for FFPE samples (40% of biopsies), indicating that the ability to detect alterations is lower for liquid biopsies. These data are consistent with the literature comparing liquid and tissue biopsy samples (14–18). Takeda et al. showed that the success rate of the NGS assay performed with FFPE samples, including 34% of biopsy specimens, was 96.7% in different types of cancer (19). The success rate was higher than that observed in previous studies. The authors suggest that the high success rate could be attributed to the fact that they assessed the tissue prior to shipping. The detection rate was lower in a study by De Falco et al. (20), who reported that 84 of 122 (68.85%) tumor samples were detected successfully. They explained that the success rate was influenced by the type of FFPE sample (FFPE block *vs.* FFPE slides), the origin of the sample (surgery *vs.* biopsy), and the time of fixation (<5 years *vs.* ≥5 years) (20).

The most commonly altered genes by genetic variation of class 4-5 and VUS in this study were consistent with those published in the literature, with TP53 as the most common gene, followed by RAS, PIK3CA, and CDKN2A/B (15, 16, 19, 20). In the present cohort, at least one actionable alteration was detected in 75% of the patients regardless of tumor type, including 62% of patients tested by liquid biopsy and 88% of those tested with an FFPE sample. Actionable alterations were found in 52% of the 191 patients included in the study by Gouton et al. who underwent a FoundationOne Liquid CDx test regardless of tumor type (21). Gouton et al. included common tumor types such as lung (46%), melanoma (11%), breast (10%), and pancreatic cancers (21). A recent French study that compared the results of FMI analysis between matched FFPE and liquid samples showed that the number of actionable alterations was identical in 42% of cases; in the remaining cases, the number of actionable alterations detected was higher in tissue (35%) than in ctDNA (23%) samples (9). Our rates of detection of actionable alterations using liquid biopsy and tissue samples was comparable with that reported in the literature (9).

We showed that among the most represented cancers, glioma testing failed in 22% compared to lung cancer samples (16% of cases) and breast cancer (14%). Moreover, we showed that Actionable class 4–5 variants were found only in 42% of gliomas compared with 66% of lung cancers, 90% of colorectal or prostatic cancers, and 96% of breast cancers. The central nervous system is separated from the rest of the body by the blood-brain barrier (BBB), and unlike other tumors, glioblastomas release few circulating tumor cells and ctDNA, especially when the BBB is intact, i.e., not yet affected by the lesion, or when the impermeability has not been modified by the intrinsic biology of the tumor (22–24). Data in the literature report highly variable rates of ctDNA detection, indicating that this is a rather unreliable method in terms of sensitivity and reproducibility (22–24). So we propose a study dedicated specifically on brain tumors to evaluate the analysis of molecular screening in exosome to increase the dectection of Actionable class 4–5 variants and decrease the testing failure. (ExoGLIE: Clinical relevance of the detection of molecular abnormalities in glial tumor exosomes - 87RI22_0011).

Previous retrospective and prospective studies indicate that treatment changes resulting from detection of actionable mutations happen in a small proportion of patients (15, 25). In our series, the molecular testing results led to treatment changes in 13 patients (8.6%). This rate is similar to that reported previously (19, 26) but slightly lower than that of clinical trials specifically designed to treat patients with targeted therapy (10, 27), in contrast to our study that included all patients. Our patients who were eligible for the test had a good Eastern Cooperative Oncology Group performance status (0 or 1); 87.6% of them had received at least two lines of treatment and had glioblastoma, lung cancer, breast cancer, or colorectal cancer. Of 69 MMTB assessments performed, 60 resulted in proposed medical treatments. Several studies showed the impact and importance of MMTB analysis in treatment decisions, patient outcomes, and clinical trial enrolment (28–30). Although FMI report offers the possibility to participate in a clinical trial available worldwide, few patients are finally included in french clinical trial centers. In most cases, patients are not eligible for inclusion because a study is no longer open for inclusion for a particular cohort or the patient does not meet all the conditions for inclusion in the study (target lesions, contraindications). This illustrates the difficulties encountered by physicians practicing in a center such as the University Hospital of Limoges, which does not currently have a labeled early phase center (CLIP, labeled by the National Cancer Institute), to include their patients in early phase clinical trials Digital platforms are therefore emerging to address this problem to improve and speed up patients’ inclusion in clinical trials (e.g: https://www.klineo.fr/ or https://www.oncoclic.fr/). These solutions will help patients access easily and quickly the latest drugs from research. In addition, the low rate of patients who are eligible for targeted therapy, may also be due to the fast clinical deterioration of patients, which limits enrollment in clinical trials, early access to therapies or to compassionate treatment. This supports the need to perform tumor sequencing as early as possible in the course of the disease to maximize the benefit window of genomics-guided therapy. In general, the F1CDx is performed at diagnosis in 20% of patients, and after first-line therapy in 50% of patients (20). Data from the literature show that repeated biopsies during tumor progression, in particular after the administration of treatments that can induce significant selection pressure, are essential to assess tumor evolution and identify resistance mechanisms (31–34). Thus, it is necessary to perform genomic analysis “early and often” during treatment. The other explanation is the inclusion of a greater number of brain tumors, for which the treatment options are limited. The patients in the present cohort had on average received extensive pre-treatment with at least two lines of treatment (20), except in the case of brain tumors which were included earlier. This is consistent with the report by Gouton et al., in which the median prior lines of systemic therapy was 3 (range, 0–10) (21).

In this study, administration of genotype-directed therapy had no impact on the patient’s OS (13 months *vs.* 14 months, p = 0.95, HR = 1.04). The OS results for the patients in the present study should be interpreted with caution because the study was not randomized. However, other studies designed to include patients in clinical trials also showed no increase in OS with matched treatment (8, 21, 35). For example, Gouton et al. showed that in patients treated according to F1LCDx test results, there was no difference in the clinical outcome between those receiving molecularly matched treatment and those receiving non-molecularly matched treatments (16). The SHIVA trial, a randomized basket trial designed to compare matched targeted drugs with conventional chemotherapy in patients with advanced solid tumors, failed to show an improvement in survival or response associated with genome‐based targeted therapy (36). A recent study showed an increase in the median progression-free survival (6.5 months *vs.* 3 months *vs.* 3 months *vs.* 4 months for ESCAT I, II, III, and IV, respectively, p = 0.0125) without an increase in OS (10).

Although this strategy does not increase survival, the NGS results increase the rate of inclusion in clinical trials of experimental treatments (8, 35). For example, Coquerelle et al. showed an increase in clinical trial participation from 5% before NGS to 28% after NGS analysis (35). In any case, these inclusions allow the accumulation of data on the impact of molecular alterations in the response to therapies, the understanding of molecular oncogenic mechanisms and the validation or not of new biomarkers. The strength of the present study is the inclusion of a large sample obtained from routine screening in different cancer types in real life, in a university hospital that takes care of cancer patients in a rural department where the population is older than the French average. One of the limitations of the study is that liquid and tissue biopsy tests were considered simultaneously, although the data could be evaluated separately. This also represents the situation in current practice, in which patients may not be able to undergo further biopsies. Most studies are limited to tissue samples or liquid biopsy samples from specific cancer types.

Another limitation of this study is that it was conducted in a single center, which is associated with bias and limitations; however, the aim was to evaluate local practices and to highlight the impact of the test in routine practice. Moreover, the real-world design of the present study provides data with a real clinical impact and without the selective criteria of clinical trials. Patients who underwent testing were selected by the physician, which may lead to selection bias. In addition, we did not include patients with known actionable driver alterations such as those with EGFR mutation-positive lung cancer or HER2-positive breast cancer. These patients receive targeted molecular therapy following testing with rapid companion diagnostics such as EGFR mutation testing or HER2 fluorescence *in situ* hybridization. Most clinical trials exclude brain tumours (e.g. patients under corticosteroids), so it is rare in molecular testing trials to see patients with brain cancer. Because of our interest in brain tumors (clinical and basic research), we also wanted to offer these patients access to molecular screening, which explains the higher number of brain tumors in this real-life study.

## 5 Perspectives

To the best of our knowledge, the present study is the first to include a large number of brain tumors in the analysis. These pathologies are generally excluded from clinical trials based on genomic analysis, and previous studies included few patients (10, 15, 37, 38). Therefore, we provide a particularly interesting view of this subgroup of patients with a poor prognosis that are frequently in therapeutic impasse. Even if the number of actionable alterations is less than in other tumors, it is still possible to detect them by broad panel testing. The ESMO recommends routine use of NGS for tumor samples from advanced non-squamous non-small-cell lung cancer, prostate cancer, ovarian cancer, and cholangiocarcinoma (5). ESMO acknowledges that although the patient and the physician can decide to analyze a large panel of genes, the patient needs to be informed regarding the low likelihood of benefit outside the indications defined by ESMO recommendations (5). Large panels of genes should be used only if they lead to an acceptable increase in the overall cost, drugs included (5). The introduction of NGS has increased the potential detection of mutations, which has an important impact on the diagnosis and treatment of many diseases. The impact of NGS on the budget and its cost-effectiveness compared with the standard single test approach need to be considered. This study shows the need to set up diagnostic platforms in the various reference centers in order to improve the coverage of the territory and to offer a wider range of care to patients. In addition to the classic indications for NGS, it is necessary to explore the molecular abnormalities of each patient in greater depth, and to promote the organization of multidisciplinary molecular consultation meetings by integrating these data into the clinical and global context of the patient. The growing contribution of bioinformaticians will be essential, as will the existence of platforms making it possible to know in real time the clinical trials available. In the future, it will be necessary to better identify patients who can benefit from molecular screening in order to optimize inclusion in trials.

## 6 Conclusions

This real-world study highlights that a center with multidisciplinary molecular tumor counseling and NGS screening system can achieve results comparable to large centers in terms of patient inclusion in clinical trials. Optimization of the screening and testing methods resulted in a data acquisition success rate and patient treatment rates similar to those obtained in larger centers. The main limitations to implementing genome-guided therapy were the clinical condition of the patient and access to drugs. Early and serial in-clinic sequencing, as well as expanded access to targeted agents and genomics-guided early phase clinical trials, can increase. Further studies focusing on the subgroup of patients with brain tumors could be of value for comparing the results of liquid and tissue biopsies. In the era of precision oncology, it is essential to identify new methods or technologies for patient selection and to define the right time to propose NGS.

## Data availability statement

The original contributions presented in the study are included in the article/**Supplementary Material**. Further inquiries can be directed to the corresponding author.

## Ethics statement

Ethical review and approval was not mandatory for the study on human participants in accordance with the local legislation and institutional requirements., however, a committee has to validated the project (N°583-2022-239).

## Author contributions

Conceptualization: AC, KD, ED. Methodology: AC, KD, ED. Formal analysis: SD, ED. Writing - original draft preparation: SP, SD, AP, LD, KD, ED. Writing - review and editing: SP, SD, AP, LD, FA, MY, TD, AV, TE, YS, VB-L, JP, LV,FT, AC, FL, KD, ED. validation: SP, SD, AP, LD, FA, MY, TD, AV, TE, YS, VB-L, JP, LV, FT, AC, FL, KD, ED. supervision: KD, ED. All authors have read and agreed to the published version of the manuscript.
