# Supplementary material for: Clinical management of molecular alterations identified by high throughput sequencing in patients with advanced solid tumors in treatment failure: Real-world data from a French hospital
Source: Front Oncol. 2023 Feb 27;13:1104659. doi: 10.3389/fonc.2023.1104659 (PMC10009270; doi:10.3389/fonc.2023.1104659)

Clinical management of molecular alterations identified by high throughput sequencing in patients with advanced solid tumors in treatment failure: real-world data from a French hospital

Supplementary Material

# Supplementary Figures and Tables

## Supplementary Table 1: Genes most altered by genetic variation of class 4-5 and variants of unknown significance (VUS) among representative cancers from our cohort

 Cancers for which more than five patients had available sequencing results were analyzed. Genes showing class 4–5 and VUS genetic alterations in at least four patients with the most represented cancers in our global cohort (glioma, lung, breast cancers), and those showing class 4–5 and VUS genetic alterations in at least two patients with the least frequent cancers (colorectal, prostate, pancreatic, ovarian, head and neck, gastric cancers) were selected.

Abbreviations: Nb.: number of patients with genetic alterations; Freq.: frequency (in %).

## Supplementary Table 2: Altered genes common to or associated with several or only one cancer among representative cancers from our cohort (listed in Supplementary Table 1)

Cancers for which more than five patients had available sequencing results were analyzed. The numbers indicate the frequency of alterations among cases of cancer. The colors represent the following: yellow = frequency <30%; orange = frequency ≥30% and <50%; and red = frequency ≥50%. Class 4–5 alterations and variants of unknown significance (VUSs) were considered for this analysis.

## Supplementary Table 3: Association of FoundationOneCDx panel genes with KEGG pathways

# Supplementary Figure

## Supplementary Figure 1

A) Prognostic value of clinical and genetic parameters was estimated using univariate and multivariate Cox regression models, and expressed with their hazard ratio (HR) and 95% confidence interval (CI). Parameters with p value inferior to 0.2% in univariate Cox analysis were selected for multivariate Cox analysis. Number of individuals composing different group was indicated for each tested parameters.

B) Forest plot of significantly independent prognostic parameters identified in multivariate Cox regression analysis.


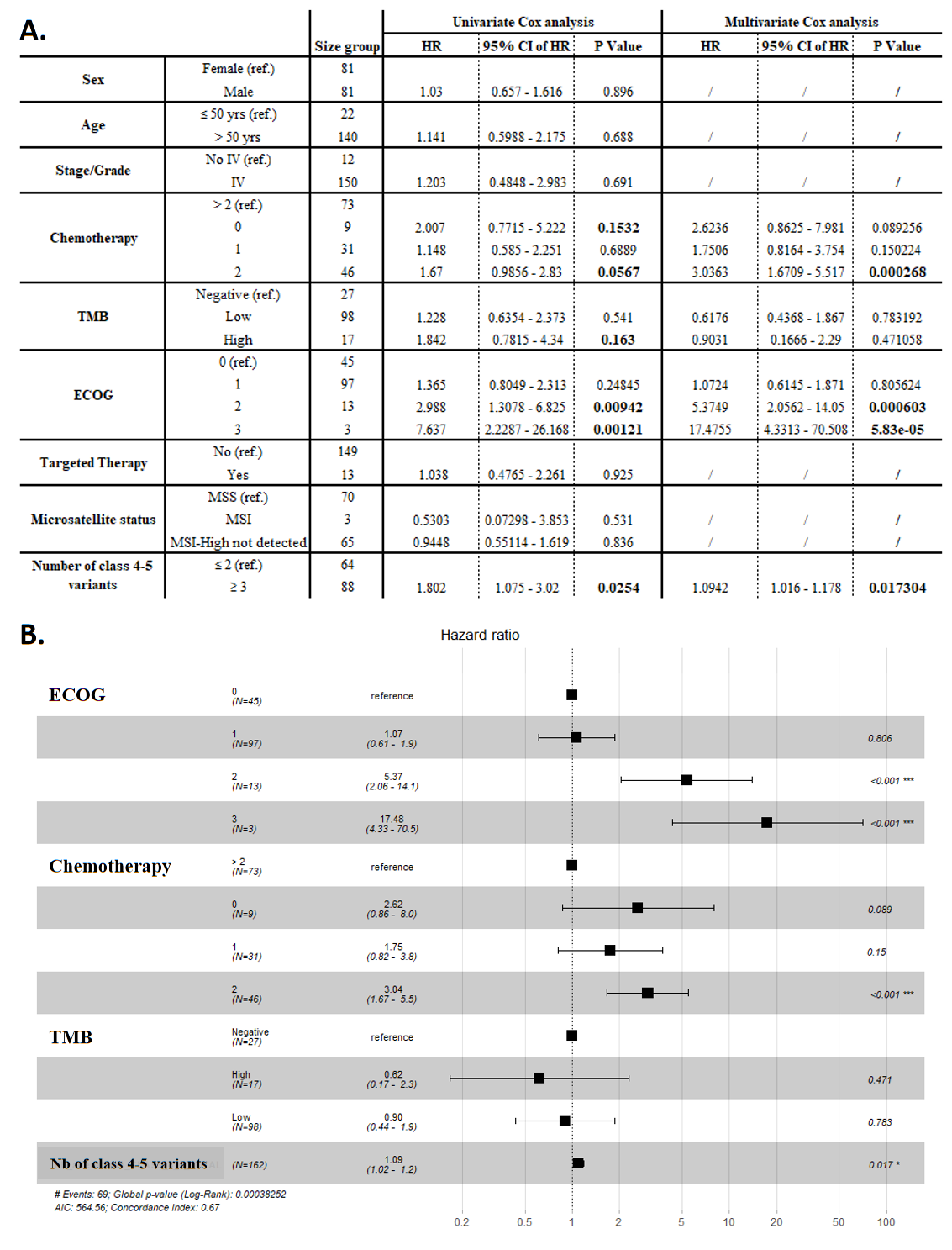

Supplement: Supplementary file 1 [file DataSheet_1.docx]
